# Case of Unusual Morbid Condition of the Heart, Discovered after Death

**Published:** 1846-06

**Authors:** C. F. Hodson

**Affiliations:** Surgeon, Bishop Stortford


*Case of Unusual Morbid Condition of the Heart, discovered after Death.* By C. F. HODSON, Esq., Surgeon, Bishop Stortford.—A blacksmith, aged twenty-seven, came into the Infirmary of this Union House a short time since, rapidly sinking. He was jaundiced, and much emaciated, complaining of diarrhœa, and urgent dyspnœa. The former was easily relieved, but the latter continued until his death, which occurred shortly after his admission. Upon examination after death, the liver was found to be in the first stage of cirrhosis; the stomach tolerably healthy, the lungs particularly so; the pericardium contained about seven ounces of serum. The right side of the heart presented concentric hypertrophy of the left ventricle, with "vegetation" disease of the semilunar valves. Contained in the chamber of this ventricle, there were from fifty to sixty globular membranous sacs, filled with a puriform fluid, and varying in size from a pea to a small nut. They were slightly adherent to the walls of the ventricle by a small portion of their surfaces between the columnæ carneæ, the smaller ones being thus almost hidden, and the larger projecting into the chamber. I have ascertained that the man had been ill for several months, and was of exceedingly intemperate habits.—*London Lancet.*

